# Predatory Potential of *Chrysoperla carnea* and *Cryptolaemus montrouzieri* Larvae on Different Stages of the Mealybug, *Phenacoccus solenopsis*: A Threat to Cotton in South Asia

**DOI:** 10.1673/031.012.14701

**Published:** 2012-12-15

**Authors:** Hafiz Azhar Ali Khan, Ali H. Sayyed, Waseem Akram, Sabtain Raza, Muhammad Ali

**Affiliations:** ^1^ Department of Entomology, University College of Agriculture, Bahauddin Zakariya University, Multan, Pakistan; ^2^ Institute of Molecular Biology and Biotechnology, Bahauddin Zakariya University, Multan, Pakistan; ^3^ Department of Agri-Entomology, University of Agriculture, Faisalabad, Pakistan

**Keywords:** cotton mealybug, developmental duration, fecundity

## Abstract

The outbreaks of mealybug, *Phenacoccus solenopsis* Tinsley (Homoptera: Pseudococcidae), have created problems to cotton crops in South Asia in the recent years. To control this menace, predatory potential of *Chrysoperla carnea* and *Cryptolaemus montrouzieri* larvae were investigated under laboratory conditions (27 ± 5° C and 65 ± 5% RH). The experiments were conducted in no choice (only first, second, or third instar larvae of mealybug were offered at a time) and choice (first, second, and third instar larvae were offered simultaneously) feeding tests. Both predators had high consumption rates, with *C. montrouzeiri* being the most voracious feeder. In the no choice feeding tests, third instar larvae of *C. montrouzeiri* devoured the highest mean number of first instar *P. solenopsis* (439.38) In the choice feeding tests, a similar number of first instar nymphs (410) were consumed. In both feeding tests, *C. carnea* devoured relatively fewer numbers of *P. solenopsis* than *C. montrouzeiri*. Manly's preference index suggested that the both predators preferred first instar nymphs of *P. solenopsis* over second or third instar nymphs. Furthermore, studies on developmental rate and fecundity revealed that first instar nymphs of *P. solenopsis* significantly reduced development time but increased the fecundity of both predators.

## Introduction

Since 2005, *Phenacoccus solenopsis* Tinsley (Homoptera: Pseudococcidae), a possibly introduced mealybug, has been causing severe damage to cotton in Pakistan and India (Hodgson et al. 2008), and also to many other plant species of crops, weeds, ornamentals, and medicinal plants (Arif et al. 2009). Exotic pests, especially those that are polyphagous with a wide host range, establish themselves easily in the introduced countries because of the absence of their native, naturally occurring predators, parasitoids, and pathogens. Moreover, pests like *P. solenopsis* establish and spread more easily than many other insect species due to a waxy coating on their dorsal side, which protects them from insecticides, a high reproductive rate, and the propensity to spread quickly through natural carriers such as plant products, wind, water, rain, birds, human beings, and farm animals. Mealybugs suck sap through phloem tissues, causing leaves to turn yellowish and fall off. The pest also secretes honeydew, which allows sooty mold to grow and hampers photosynthetic process (Al adwai et al. 2006). They also seem to be able to become dormant on inert material for considerable periods of time under unfavorable conditions. Furthermore, problems with insecticide resistance and non-target effects on natural enemies make chemical control a less desirable control option to combat the *P. solenopsis*. To lessen the intensive use of pesticides, there is a need to establish an integrated pest management program (Van den Bosch et al. 1982) for *P. solenopsis*. The integrated pest management concept proposed by Stern et al. (1959) advocates both chemical and biological control in agricultural systems. However, biological control agents are difficult to maintain when pesticides are applied to control key pests because natural enemies are often more sensitive to insecticides compared with the pests.

Multiple natural enemy species can cause interactions in predators and prey by reducing or increasing predation risk for the prey (Sih et al. 1998), and this interaction may increase or decrease the equilibrium level of the prey (Losey and Denno 1998). The application of predators for a successful biological control program could be controversial, due to their potential to prey on other biological control agents and non-target species (Symondson et al. 2002). In such a biological control where multiple agents are released for colonization, the interactions among the biological control agents could determine the success of the program (Denoth et al. 2002).

Interest in using beneficial predators as a component of integrated pest management programs for field and horticultural crops has recently increased, as growers seek alternatives to insecticides for managing insect pests. The larvae of *Chrysoperla* spp. are among the most efficient predators of many important agricultural insect pests (Lingren et al. 1968), particularly *C. carnea* (Stephens) (Neuroptera: Chrysopidae), which is the most abundant species in the genus (Van den Bosh and Hagen 1966). They can inhabit many diverse agroecosystems, and they are easily mass reared (Rajakulendran and Plapp 1982). The larvae of *C. carnea* are voracious and generalist predators, while adults only feed on nectar and pollen (Tauber et al. 2000). *C. carnea* has effectively been used in Pakistan and thus has been proved to be a voracious predator of cotton mealybug (Sattar et al. 2007). Besides *C. carnea*, *Cryptolaemus montrouzieri* Mulsant (Coleoptera: Coccinellidae) is also known as a voracious feeder of various mealybug species (Hodek and Honek 2009) and has successfully been applied to control *Planococcus citri* (Risso) and *Phenacoccus gossypi* (DeBach and Schlinger 1964). However, *C. montrouzieri* is not a common predator in Pakistan, so Pakistan has had to import the beetle from the USA. *C. montrouzieri* is known to appear at later stages of cotton mealybug infestation, but has successfully been used against several species of cotton mealybug in different countries (Karte 2008)

The combination of predators produced varying degrees of success, both in the fields and greenhouses. Failures may be the result of unfavourable environmental factors, incompatible pest management practices, and antagonistic interactions among the biological control agents. *C. montrouzieri* has the potential of interfering with the biological control of the mealybug (Sengonca and Yanuwiadi 1994). The present study was therefore designed to investigate the predatory potential of *C. carnea* and *C. montrouzieri* against *P. solenopsis* in the laboratory. Furthermore, we explored the potential of competition or interference between the two biological control agents. The objectives of the studies were achieved by assessing the predatory potential of *C. carnea* and *C. montrouzieri* at specific larval stages in choice and no choice feeding tests, and finding the differences in the predatory potential of both species at each larval instar.

## Materials and Methods

### Insects

The *P. solenopsis* was collected from the field and maintained on cotton plants in the laboratory at 27 ± 2° C, 65 ± 5% RH, and 16:8 L:D. *C. carnea* were obtained from the Bio-Control Laboratory of the University of Agriculture, Faisalab ad, where they were maintained on *P. solenopsis* at 27 ± 2° C, 65 ± 5% RH, and 16:8 L:D for over six years. *C*. *montrouzieri* were obtained from CABI South Asia, where they were maintained on *P. solenopsis* for over two years. Before the start of experiment, both predators were maintained on *P. solenopsis* for at least five generations at 27 ± 2^°^C, 65 ± 5% RH, and 16:8 L: D.

### Consumption rate

Three larval instars of both predator species were used to study the consumption rate of *C. carnea* and *C. montrouzieri*. The experiment was carried out using free choice and no choice feeding tests. In the free choice feeding tests, a single larva of a specific instar of *C. carnea* and a single larva of a specific instar of *C. montrouzeiri* were released separately into separate plastic jars (20 × 10 cm) lined with muslin cloth for aeration. A mixture of 500 nymphs of each instar (first, second, and third) of *P. solenopsis* was introduced into each jar, and fresh cotton leaves were provided for *P. solenopsis*. Daily consumption was calculated by subtracting the number of *P. solenopsis* individuals left from the number of individuals brushed in the jar. The numbers of *P. solenopsis* consumed were counted every 24 hours until the end of each larval instar of predator species.

In the no choice feeding tests, the predatory potentials of both predator species were assessed at each specific larval instar by offering 500 nymphs of first, second, and third instars of *P. solenopsis* separately. Fresh cotton leaves were provided in the jars for *P. solenopsis*. Both experiments, choice and no choice, were replicated eight times.

### Development and fecundity

The effects of *P. solenopsis* on development and fecundity of *C. carnea* and *C. montrouzieri* were examined by exposing the predators to various instars of *P. solenopsis*, as described above. The time interval of developmental stages of both predators was recorded every day until pupation and adult eclosion.

After eclosion, the adults of both predatory species were paired, and each pair was kept in a separate transparent plastic jar (20 × 10 cm) at 27 ± 2° C, 65 ± 5% RH, and 16:8 L:D, where *C. arnea* were fed on a diet prepared from water, honey, and yeast (Hassan, 1975), and *C. montrouzieri* were fed on *P. solenopsis*. The number of eggs laid by both predators was counted every day until the death of females.

### Statistical analysis

Kolmogorov-Smirnov test was applied before any statistical analysis to check the normality of the data. The difference in the predatory potential of six larval instars (three instars of each predator species) in choice and no choice feeding experiments was compared by using two ways analysis of variance (ANOVA) (Analytical Software 2005). A least significant difference test (α = 0.05) was performed to separate the means when differences were significant (Winer 1991). A paired *t-*test was applied to compare the predatory potential of *C. carnea* and *C. montrouzeiri* at each specific larval instar

The preference for various instars of *P. solenopsis* was determined by calculating the Manly's preference index (αp) (Manly et al. 1972) in the choice test. Because the number of *P. solenopsis* within each replicate was reduced over time due to feeding by *C. carnea* and *C. montrouzeiri*, the modified food-depletion equation for the preference index was used, where:
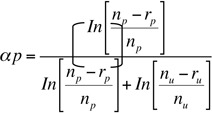

The parameters n_p_ and n_u_ were the initial numbers of *P. solenopsis* (i.e., 500), respectively; r_p_ and r_u_ were the numbers *P. solenopsis* consumed over a 24 hour period, respectively. The preferences of all instars of *C. carnea* and *C. montrouzeiri* were calculated separately. This index gives values of preferences ranged from 0 to 1, where 0.5 indicates no preference. We tested our null hypothesis of no preference for a specific instar of *P. solenopsis* with a *t-*test.

## Results

### Consumption rate

In the no choice feeding tests, the number of *P. solenopsis* consumed significantly increased at each successive predatory instar (F = 453.50; df = 5,119; *p* < 0.001). However, the number of second and third instar *P. solenopsis* consumed did not differ significantly when they were exposed to second or third instars of *C. carnea* (*p* > 0.05) (Table 1). In contrast, significant differences in consumption rate were observed between all instars of *C. carnea* when they were fed on first instar of *P. solenopsis*. No statistical difference was observed between second or third instars of *C. montrouzieri* when they were fed on second and third instar of *P. solenopsis*. Likewise, no difference was observed with all three instars of *C. montrouzieri* when they were fed on third instar *P. solenopsis* (*p* > 0.05). Consumption rate of all three instars of *C. montrouzieri* were significantly different when they were fed on first instar of *P. solenopsis* (*p* < 0.01) (Table 1).

In the choice feeding tests, significantly higher consumption of *P. solenopsis* was observed at each successive predatory instar (F = 1719.82; df = 5,119; *p* < 0.01). However, consumption of third instar *P. solenopsis* was similar throughout the larval stages of *C. carnea* and *C. montrouzieri* (*p* > 0.05). In contrast, when third instar larvae of *C. montrouzieri* were used, it consumed the highest number of *P. solenopsis* in both feeding tests (*p* < 0.01) (Table 2).

### Manly's preference index

**Figure 1.**
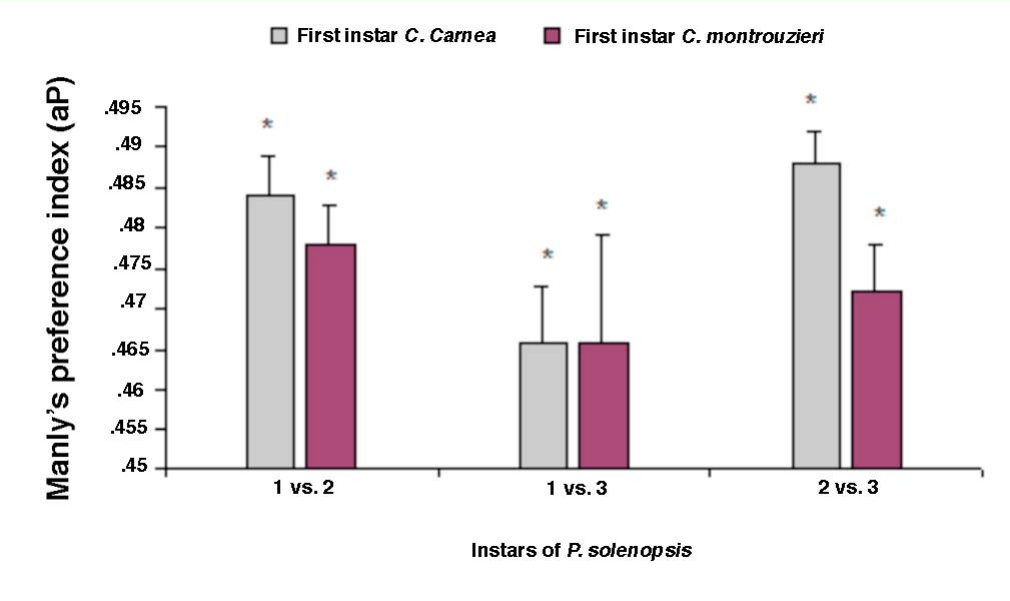
Manly's preference index (αp) of first instar larvae of *Chrysoperla carnea* and *Cryptolaemus montrouzieri* for different stages *of Phenacoccus solenopsis*. The bars with asterisks are significantly different from the predicted index of 0.5 by *t*-test. High quality figures are available online.

**Figure 2.**
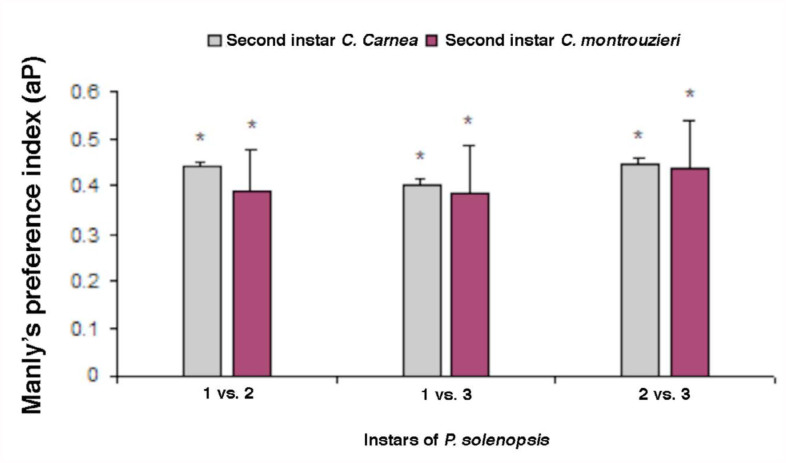
Manly's preference index (αp) of second instar larvae of *Chrysoperla carnea* and *Cryptolaemus montrouzieri* for different stages of *Phenacoccus solenopsis*. The bars with asterisks are significantly different from the predicted index of 0.5 by *t*-test. High quality figures are available online.

**Figure 3.**
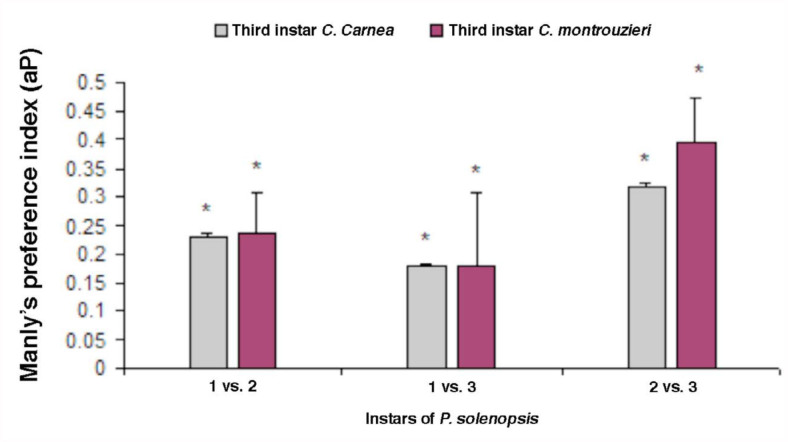
Manly's preference index (αp) of third instar larvae of *Chrysoperla carnea* and *Cryptolaemus montrouzieri* for different stages of *Phenacoccus solenopsis*. The bars with asterisks are significantly different from the predicted index of 0.5 by *t*-test. High quality figures are available online.

When younger vs. older instar (i.e.’ first vs. second instar; first vs third instar; second vs third instar) of prey were offered simultaneously, both predator species consumed all the larval stages of prey, but preferred younger instars (Figures 1–3) Index values > 0.5 represent preference for older instars (positive switching), while those < 0.5 represent preference for early instars of prey (negative switching) (Blackwood et al. 2001). The first instar larvae of *C. carnea* (t = 12.4; df = 4; *p* = 0.002) showed marked preference for first instar nymphs of *P. solenopsis* over second or third instar (t = 44.79; df= 4; *p* = 0.000; Figure 1). Similar results were observed for *C. montrouzieri* (Figure 1). Likewise, second instar of *C. carnea* and *C. montrouzieri* also showed significantly greater preference for first instar nymphs of *P. solenopsis* over second instar (t = 35.36; df= 4; *p* = 0.000; t = 21.98; df = 4; *p* = 0.000 respectively) and third instar (t = 99.57; df= 4; *p* = 0.000; t = 50.13; df= 4; *p* = 0.000; Figure 2). When the third instar of the predators were compared for predation potential for various stages of *P. solenopsis*, the predators preferred younger nymphs over older (Figure 3). In other words, both predators showed negative switching as both preferred younger instars.

### Development and Fecundity

Prey stages also had a significant effect on development time. Development time increased significantly when third instar nymphs of *P. solenopsis* were offered to predators. The development time for *C. carnea* was 19.9 days, but it was 20.3 days for *C. montrouzieri* when offered third instar of *P. solenopsis* (F = 10.72; df = 2,12; *p* = 0.002: F = 13.62; df = 2,12; *p* = 0.001; respectively) (Table 3).

Different instars of *P. solenopsis* had no significant impact on pre-ovipositon and post-ovipositon or longevity period of adult predator females. In contrast, various instars of *P. solenopsis* significantly influenced the oviposition period of *C. montrouzieri* (Table 4). The fecundity differed significantly when predators were fed on different prey stages. Higher numbers of eggs were laid by *C. carnea* and *C. montrouzieri* (310 and 338 respectively) when their immature stages were raised on first instar nymphs of *P. solenopsis*.

## Discussion

Consumer-resource relationships could play an important role in ecology, influencing both dynamics of populations and the flow of energy through food webs. Predators can impact prey populations directly through prey consumption (Peckarsky et al. 2008). Consumption of herbivores has a known cost, so it is important to know the number of prey killed when determining the effects of predators on prey population dynamics (Messina and Sorenson 2001). Our results suggest that both predators show strong predatory potential against *P. solenopsis*, with *C. montrouzeiri* being the most ravenous feeder. Moreover, prey stages also had a considerable effect on consumption rate, development, and fecundity. Our findings are similar to those of Sattar et al. (2007), who reported that the third instar larvae of *C. carnea* had voracious behavior compared to second or first instar in free choice feeding tests. The second and third instars of *P. solenopsis* were less preferred by the predator species compared to the first instar, which was similar to a previous study (Copland et al. 1985; Gautam and Tesfaye 2002). The most probable reason for preference of first instar larvae could be due to small size. A steady increase in predation was recorded with the progression of larval developmental stages. The difference in the ability of the predators to recognize suitable prey may be related to their morphological differences (Dixon 2000), the changes in the chemical and physical characteristics in prey (Omkar et al. 2004), and the combination of these factors. The mechanisms by which the predators locate and recognize suitable prey instar are largely unknown. Further studies are therefore required to determine these mechanisms and their roles in prey discrimination. In contrast, results of our studies suggest that *C. montrouzieri* could be very effective at all stages against *P. citri* when used in combination with predators or parasitoids (Copland et al. 1985). Similarly *C. montrouzieri* has previously been shown to successfully suppress the population of *Maconellicoccus hirsutus* in the Caribbean (Kairo et al. 2000).

*C. montrouzieri* oviposition period was 45% longer when reared on first instar of *P. solenopsis* compared to third instar. Similarly *C. montrouzieri* female laid a significantly higher number of eggs when reared on first instar *P. solenopsis*. Prey size also increased fecundity of *C. carnea*, but there was no observed effect of prey size on oviposition period or longevity (Table 4). Similarly, Horton et al. (1998) showed that the adults of *Perillus bioculatus* fed fourth-instar larvae took 65% longer to develop and 30% longer to develop to adulthood than those reared on first-instar prey, affecting several fitness components. The low fecundity shown by both predators when reared on late instars of *P. solenopsis* could be due to the fact that the late instars were able to escape from *C. montrouzieri* or *C. carnea*, and therefore the predators were under-fed at immature stages. The results of this study must be considered with utmost care when comparing to field conditions. Even though the prey was confined in the laboratory conditions, the later instar nymphs still managed to escape the predators. In the field, the nymphs of *P. solenopsis* could have even more freedom to move.

Our data further suggest that the second and third instar *C. montrouzieri* or *C. carnea* generally attack smaller prey compared with fourth instar larvae, which is consistent with the hypothesis that the predators usually select such stages of prey that could maximize their fitness. Our experiment therefore suggests that both the predators will have greater impact on *P. solenopsis* at earlier stages than when the population has developed to fourth instar. The larger prey can usually weaken the invertebrate predator species and overcome the predator, resulting in a reduced predator population. For example, consumption rate has been shown to decline with increased prey size in aphidophagous coccinellid beetles and anthocorid bugs (Dixon and Russel 1972).

Differences between different instars of *C. montrouzieri* or *C. carnea* developmental rates, preoviposition, ovipostion periods, longevity, and fecundity should reduce stage-structure synchrony between the predator and prey populations. Such stage-distribution asynchrony could limit the ability of the predator. For this reason, the phenology of the predators' first instars feeding on *P. solenopsis* is likely to be affected more vigorously than later instars. If *P. solenopsis* first instars are not available in the field, then it seems quite possible for the predators’ juveniles to complete their entire immature stage development on later stage *P. solenopsis*. The predator populations probably grow slowly, if at all, at these times, which may account for their inability to respond numerically to changes in prey density or to regulate *P. solenopsis* populations, a situation further exacerbated by the dearth of all sizes of prey during the pupation and preoviposition adult phases of *L. decemlineata* cohorts. Third instar *P. solenopsis* nymphs are responsible for the majority of the damage. Thus, the predators are particularly ineffective at controlling third instar *P. solenopsis*. The impact of large prey on the predator consumption rates and development time reported here underscore the need to synchronize augmentative releases of the predators to the phenology of the most vulnerable stages (eggs and first instar nymphs) of *P. solenopsis*.

The decline in prey capture success with increased *P. solenopsis* instar seems to constitute a body size refuge from *C. carnea* and *C. montrouzieri* predation. Theoretical work has shown that prey refuges can destabilize predator-prey interactions by causing prey outbreaks (Collings 1995). The evidence presented here for both predators suggests that prey size refuges can destabilize predator-prey interactions by reducing predator consumption and developmental rate, limiting their capacity to numerically respond and to regulate prey populations. Other predators with large prey may also be unable to regulate their prey, and predator-to-prey size ratios should be considered in the selection of biological control agents.

**Table 1.**
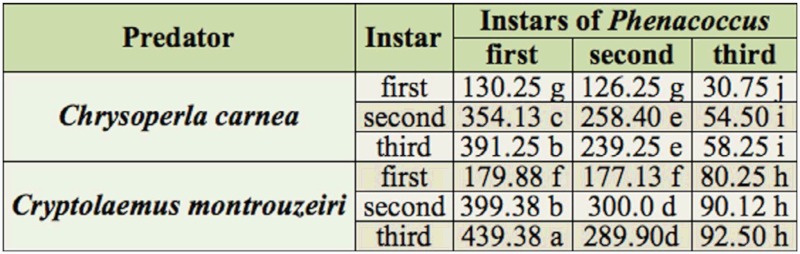
No choice predation by *Chrysoperla carnea* and *Cryptolaemus montrouzieri* on different stages *of Phenacoccus solenopsis*. The values are the mean number of *P. solenopsis* consumed. Means followed by the different letters are significantly different from each other at *p* value < 0.05 (two way ANOVA and least significant difference 5%).

**Table 2.**
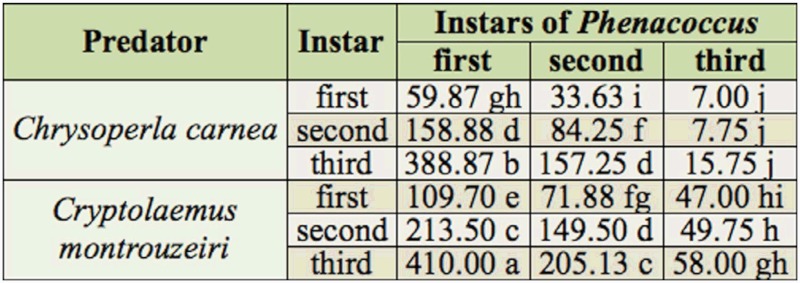
Free choice predation by *Chrysoperla carnea* and *Cryptolaemus montrouzieri* on different stages *Phenacoccus solenopsis*. The values are the mean number of *P. solenopsis* consumed. Means followed by the different letters are significantly different from each other at *p* value < 0.05 (two way ANOVA and least significant difference 5%).

**Table 3.**
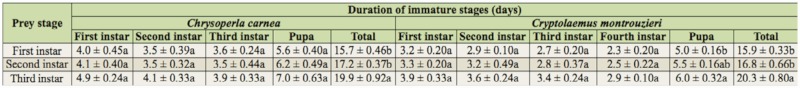
Mean duration of immature stages of *Chrysoperla carnea* and *Cryptolaemus montrouzieri* feeding on different stages of *Phenacoccus solenopsis*. The values are the mean duration of different immature stages (± SE). Means having similar letters within a column are not significant at *p* < 0.05 (ANOVA and least significant difference 5%). Total = first instar to adult.

**Table 4.**
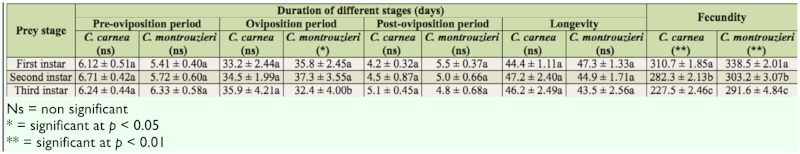
Fecundity and longevity of *Chrysoperla carnea* and *Cryptolaemus montrouzieri* females developed from larvae fed with different stages of *Phenacoccus solenopsis*. The values are mean duration of different immature stages (± SE). Means having similar letters within a column are non significant at *p* < 0.05 (ANOVA and least significant difference 5%). Fecundity = number of eggs per female.
